# Differences in nutritional status and level of physical activity among adolescents living in urban and rural areas of Montenegro - national study

**DOI:** 10.1186/s12889-024-18402-3

**Published:** 2024-03-26

**Authors:** Erol Vrevic, Pavle Malovic, Dragan Bacovic, Danilo Bojanic, Aldijana Nokic

**Affiliations:** Faculty for Sport and Physical Education, University of Montenegro, Podgorica, Montenegro

**Keywords:** Adolescence, Obesity, Overweight, Physical activity, Place of residence

## Abstract

**Background:**

Nutritional status and physical activity are important factors for adolescent health. These factors may vary by the place of residence. This study aims to assess the nutritional status and physical activity levels, as well as their variations by the place of residence.

**Methods:**

The sample consisted of 1503 adolescents (46.3% male; 53.7% female), with a mean age of 15.7 ± 0.7 years. Nutritional status was assessed using Body Mass Index (BMI), Waist-to-Height Ratio (WHtR), and Body Fat Percentage (BF). Physical activity levels were assessed using the PAQ-C questionnaire. Differences in nutritional status among adolescents living in urban and rural areas were analyzed using Chi-square test (*p* ≤ 0.05), while differences in physical activity levels were analyzed using an independent samples t-test (*p* ≤ 0.05).

**Results:**

In terms of BMI, 14.7% of males were overweight and 10.1% were obese, while 12% of female adolescents were overweight and 3.1% were obese. There were no differences in nutritional status among adolescents living in urban and rural areas (BMI, WHtR, BF). Male adolescents in rural areas had significantly higher physical activity levels compared to urban areas (*p* = 0.032). They showed higher activity scores during a spare time (*p* = 0.002), physical education classes (*p* = 0.002), little breaks (*p* = 0.007), and lunchtime (*p* = 0.034). Female adolescents showed significance only in spare time activity (*p* = 0.020).

**Conclusion:**

The differences in nutritional status among adolescents living in urban and rural areas were not found. However, male adolescents living in urban areas showed lower physical activity levels than their rural counterparts. Strategies should be implemented to promote physical activity among adolescents in urban areas, and attention should be given to the further urbanization process to create improved conditions for engaging in physical activity.

## Background

Adolescence represents a crucial period of life and is essential for the development of socially responsible individuals [1], and poor health during adolescence can have long-term consequences [2]. Approximately 35% of health problems are believed to originate in adolescence [3]. Therefore, it is crucial to consider all components that contribute to the development of socially responsible and functional individuals, especially those that can influence their health. However, there is limited attention given to the effects that can have a relationship with the health of adolescents [4], including nutritional status and levels of physical activity.

Overweight and obesity represent significant contemporary issues that can negatively impact the health of adolescents. Considering adolescents with overweight and obesity are at a high risk of facing these problems throughout their lives [5], it is clear that timely intervention is crucial. Unfortunately, the current practice shows a different reality, as there are currently 390 million overweight and obese children and adolescents aged 5–19 worldwide [6]. When discussing Montenegrin adolescents, data from the 2018 national study indicate that 15.2% of male adolescents and 9.7% of female adolescents in Montenegro were overweight or obese [7]. Meanwhile, the latest data from the 2023 national study, obtained from female adolescents, show that 15.4% of adolescent girls were overweight or obese [8]. Overweight and obese adolescents are at a higher risk of developing diabetes, cardiovascular diseases, cancer, hypertension, and many other chronic non-communicable diseases [9]. Moreover, overweight and obesity are negatively associated with the physical and mental well-being of adolescents, which can significantly impact their growth and development [10]. In the United States alone, obesity is estimated to be the cause of up to 365,000 deaths annually [11]. Additionally, data from 2010 revealed that excess weight and obesity contributed to 3.4 million deaths [12], meaning that this issue accounted for approximately 9,315 deaths per day, or 6 deaths every minute. Furthermore, recent economic data indicates that overweight and obesity cost the United States healthcare system over $100 billion annually [13] (cited in [9]).

Physical activity plays a significant role in maintaining the health of adolescents and preventing overweight and obesity [14], while according to WHO data [15], adolescents who engage in a minimum of 60 min of moderate to vigorous physical activity have no risk of overweight and obesity. The benefits of physical activity are numerous, including improvements in physical fitness (including cardiorespiratory and muscular fitness), maintenance of normal blood pressure, dyslipidemia, glucose, insulin resistance, better bone health, cognitive outcomes, mental health, and more [16]. Physical activity has proven as excellent for preventing non-communicable diseases such as cardiovascular diseases, cancer, and diabetes. Additionally, it influences the enhancement of cognition, learning, and reasoning skills, as well as fostering the healthy growth and development of youth [17]. Review study [18] has shown the positive impact of physical activity on depression and anxiety, mood enhancement, self-esteem, reduced susceptibility to stress, etc. Notably, physical activity also influences the elevation of plasma brain-derived neurotrophic factor, mitigating the toxicity of amyloid-beta associated with the progression of Alzheimer’s disease [19]. It is noteworthy that physically inactive individuals have a 20–30% higher risk of mortality compared to those who are physically active [17]. However, despite all the benefits of physical activity, its engagement is decreasing among adolescents [20]. Global study [21], findings encompassing a sample of 1.6 million adolescents indicate that as much as 81% do not meet the basic guidelines for physical activity, while in Asia, only 12.8% of adolescents engage in at least 60 min of physical activity per day for five or more days a week [3].

It is well known that the place of residence conditions the lifestyle of a society [22] and can have relationship with the nutritional status and level of physical activity. Differences in these components have been observed among adolescents living in urban and rural environments [23]. Although obesity was previously associated with the urbanization trend, a global study [24] shows that from 1985 to 2017, more than 55% of the global increase in BMI, and in some low- and middle-income regions even more than 80%, was due to its progressive rise in rural areas. Furthermore, the same study suggests that individuals living in rural environments may engage in less physical activity due to various reasons such as a lack of sports facilities, organized physical activities, increasing use of machinery for agricultural and other work, reduced physical movement due to developed infrastructure and availability of transportation, easier access to phones and computers in rural areas, leading to an increase in sedentary lifestyles, and so on. After the mentioned global study prompted many researchers to delve into this issue, individual studies [25, 26] indicate that individuals living in rural areas exhibit lower levels of physical activity and a higher prevalence of nutritional status. On the other hand, several studies have indicated the opposite [27–29]. Based on this, it can be observed that variations in nutritional status and levels of physical activity may differ depending on countries. Assessing these variations for each country is crucial, as it can inform different strategies, such as targeted public health interventions for communities in need, prevention, and correction of nutritional status issues in areas where it is more prevalent, and influencing urban planning to create environments that encourage physical activity and healthy lifestyles in communities with lower levels of physical activity, and so forth. This is particularly important for creating an environment conducive to normal growth and development in adolescence, representing the optimal period for preventing health problems caused by overweight and obesity and lack of physical activity [30]. No national studies on the differences in nutritional status and physical activity levels in Montenegrin adolescents were found, making this study preliminary within this sample of participants. Therefore, the aim of this study is to assess differences in nutritional status and physical activity levels among adolescents living in urban and rural areas in Montenegro.

## Methods

### Participants

The sample of participants measured in this study consisted of 1,550 male and female adolescents attending the first and second grades of secondary school. The average age of the participants was 15.7 ± 0.7 years (range: 13.9–17.9 years). The inclusion criteria for participants in this study were as follows: (1) Residing in urban or rural areas of Montenegro and attending the first or second grade of secondary school; (2) Both males and females; (3) Willingness to undergo anthropometric measurements; (4) Completion of the questionnaire. Adolescents who declined to participate in the measurements or questionnaire were excluded from this study. According to the data from the School Statistics [31], there were 13,328 students attending the first and second grades of secondary school in Montenegro during the 2021/2022 academic year, which means that the measured participants accounted for 11.6% of the total number of students. The selection of participants, as well as the schools and municipalities included in the measurements, was done through random sampling. Each school and adolescent had an equal chance of being included in the sample, aiming to obtain objective data. Regarding the schools in this study, 27 secondary schools (53%) out of a total of 51 in Montenegro [31] were included, representing 14 municipalities (56%) out of the total of 25.

### Procedures

According to the temporal orientation, this study is of a cross-sectional design. In the beginning, confirmation was obtained from the University of Montenegro (03-1593/5) for research approval. Subsequently, confirmation was obtained from the directors of the high schools, whose students are the subjects of measurement, granting access to measure anthropometric variables and administer the questionnaire. Anthropometric measurements were conducted in accordance with the standards outlined in the ISAK manual [32]. The students were informed about the significance, objectives, and procedures of the study. Prior to completing the questionnaire, participants were given clear instructions on how to fill it out, and during the survey, one of the measurers was present with students to ensure greater objectivity and validity of the data. During the anthropometric measurements, the students were barefoot and wore lightweight clothing. Students were free to withdraw from the study at any time, if they wished to do so. Testing was performed in the morning by experienced measurers who were collaborators at the Faculty of Sports and Physical Education in Niksic, thus increasing the likelihood of obtaining more relevant and precise data. The measurers were also familiar with the testing procedures and data entry methods on the measurement sheets provided for each individual student.

The place of residence was defined by having students enter the name of their place of residence in a questionnaire. Based on their place of residence and with the help of the spatial urban planning solutions of each municipality where the testing was conducted, students were categorized into urban and rural areas. The spatial urban planning solutions were obtained from the respective municipal offices responsible for spatial planning. Additionally, according to the data from Monstat [33], rural areas in the territory of Montenegro are characterized by lower population density, relatively limited or no presence of administrative centers and public services, weaker infrastructure, and mainly agricultural activities. This definition also includes urban settlements with a population of less than 10,000 inhabitants.

### Measurements

#### Nutritional status

To assess the level of nutritional status, the following anthropometric characteristics were taken: body height (BH), body mass (BM), waist circumference (WC), subscapular skinfold (SS), and triceps skinfold (TS). Based on these measurements, anthropometric indices were calculated: Body Mass Index (BMI), Waist-to-Height Ratio (WHtR), and Body Fat Percentage (BF). The assessment of nutritional status was determined by calculating percentile values for each participant’s BMI according to the standards of the Centers for Disease Control and Prevention (CDC). Participants were categorized as underweight (< 5th percentile), normal weight (5th − 85th percentile), overweight (> 85th ≤ 95th percentile), or obese (> 95th percentile). WHtR, representing the ratio of waist circumference to body height, was also used as a reliable method to assess nutritional status, with a WHtR value greater than 0.5 indicating obesity [34]. Body fat percentage was calculated using the Slaughter equation, which utilizes the subscapular and triceps skinfolds [35]. Percentile values were then used to classify participants as underweight (< 5th percentile), normal weight (5th − 85th percentile), overweight (> 85th ≤ 95th percentile), or obese (> 95th percentile) [36].

#### Physical activity

To assess the level of physical activity, the standardized International Physical Activity Questionnaire for Children and Adolescents (PAQ-C questionnaire) was used. This questionnaire measures the adolescent’s self-reported level of physical activity in different settings and parts of the day on a scale ranging from 1 to 5, where a value of 1 indicates low physical activity, while a value of 5 represents high physical activity [37]. The validity and reliability of this questionnaire have been confirmed in numerous studies [38].

### Statistical analysis

The data obtained in this study were analyzed using descriptive statistical procedures, including calculation of the minimum and maximum values, mean, standard deviation and range of variation. To assess differences in nutritional status among adolescents living in urban and rural areas of Montenegro, a chi-square test was used with a significance level of *p* ≤ 0.05. Differences in the level of physical activity among adolescents living in urban and rural areas were evaluated using independent samples t-test, with a significance level of *p* ≤ 0.05. Data processing and statistical analysis in this study were performed using the SPSS software package, version 23.0.

## Results

In Table 1, descriptive parameters of the participants are presented. Out of a total of 1550 participants who underwent the measurement process, 47 participants were excluded from the study due to incomplete questionnaires or incomplete anthropometric measurements. Thus, the final sample included 1503 adolescents, comprising 696 males (46.3%) and 807 females (53.7%). Among them, 1046 adolescents (69.6%) resided in urban regions (males: 495 (32.9%); females: 551 (36.7%)), while 457 adolescents (30.4%) resided in rural regions (males: 201 (13.4%); females: 256 (17%)). The mean age of the participants was 15.7 ± 0.7 years (range: 13.9–17.9 years). The table also displays the minimum, maximum, and mean values of all collected anthropometric measures for both genders, which were used to calculate the participants’ nutritional status based on the derived indices.

**Table 1:** Descriptive parameters of the participants

Variables	Categories	Total (*n* = 1503)	Male 696 (46.3%)		Female 807 (53.7%)	
			Urban *n* = 495 (32.9%)	Rural *n* = 201 (13.4%)	Urban *n* = 551 (36.7%)	Rural *n* = 256 (17%)
Age	M ± SD	15.7 ± 0.7	15.7 ± 0.6	15.6 ± 0.6	15.7 ± 0.6	15.7 ± 0.6
	Min	13.9	14.4	14.6	13.9	14.1
	Max	17.9	17.6	17.1	17.4	17.9
BH	M ± SD	172.6 ± 9.0	179.4 ± 7.6	176.7 ± 9.4	168.0 ± 6.2	166.0 ± 6.4
	Min	147.2	147.8	154.8	152.7	147.2
	Max	204.0	204.0	195.3	189.9	186.5
BM	M ± SD	65.5 ± 12.9	71.6 ± 13.0	69.6 ± 13.2	60.7 ± 10.5	60.4 ± 10.5
	Min	39.2	40.7	44.5	39.7	39.2
	Max	134.6	113.1	108.5	134.6	117.2
WC	M ± SD	75.6 ± 9.5	79.5 ± 9.5	77.9 ± 9.4	72.4 ± 8.2	73.2 ± 9.2
	Min	43.5	43.5	61.3	53.1	57.7
	Max	123.0	118.4	105.7	117.5	123.0
SS	M ± SD	11.3 ± 5.7	10.3 ± 5.5	9.6 ± 4.7	12.4 ± 5.6	12.5 ± 6.2
	Min	4.0	4.5	4.0	5.3	4.5
	Max	48.0	44.8	32.8	48.0	40.2
TS	M ± SD	14.8 ± 5.9	12.5 ± 5.6	11.7 ± 5.1	16.9 ± 4.8	17.2 ± 6.1
	Min	4.2	4.2	4.3	5.8	5.8
	Max	56.2	37.2	29.2	45.2	56.2

Legend: n - number; M - mean; SD - Standard deviation; Min - Minimal values; Max - Maximal values; BH - Body High; BM - Body Mass; WC - Waist Circumference; SS - Subscapular Skinfold; TS - Triceps Skinfold;

In Table 2, descriptive statistics for variables assessing the nutritional status of adolescents are presented, including minimum, maximum, mean value, standard deviation, and range of variation. The table shows that males in the total sample have a BMI value of 22.2 ± 3.5, WHtR of 0.44 ± 0.05, and BF of 16.8 ± 7.5, while female adolescents have BMI values of 21.6 ± 3.4, WHtR of 0.43 ± 0.05, and BF of 25.0 ± 6.0.

**Table 2:** Descriptive statistics of nutritional status variables

Variables	Categories	Male 696 (46.3%)			Female 807 (53.7%)		
		Urban *n* = 495 (32.9%)	Rural *n* = 201 (13.4%)	Overall *n* = 696 (46.3%)	Urban *n* = 551 (36.7%)	Rural *n* = 256 (17%)	Overall *n* = 807 (53.7%)
BMI	M ± SD	22.2 ± 3.5	22.2 ± 3.6	22.2 ± 3.5	21.5 ± 3.3	22.0 ± 3.7	21.6 ± 3.4
	Range	20.4	20.1	20.5	29.6	27.8	30.7
	Min	15.2	15.6	15.2	15.0	13.8	13.8
	Max	35.6	35.7	35.7	44.6	41.6	44.6
WHtR	M ± SD	0.44 ± 0.05	0.44 ± 0.05	0.44 ± 0.05	0.43 ± 0.05	0.44 ± 0.05	0.43 ± 0.05
	Range	0.43	0.27	0.43	0.37	0.33	0.37
	Min	0.24	0.34	0.24	0.31	0.35	0.31
	Max	0.67	0.77	0.77	0.68	0.68	0.68
BF	M ± SD	17.1 ± 7.5	16.1 ± 7.6	16.8 ± 7.5	24.9 ± 5.7	25.1 ± 6.8	25.0 ± 6.0
	Range	34.6	39.4	39.4	42.5	45.36	45.36
	Min	5.6	4.0	4.0	11.6	11.1	11.1
	Max	40.2	43.41	43.4	54.1	56.4	56.4

Legend: Range - Range of variation

The nutritional status of adolescents and their differences by place of residence are presented in Table 3. Looking at the BMI values, it can be observed that 14.7% of male adolescents were overweight and 10.1% were obese, while 12% of female adolescents were overweight and 3.1% were obese. According to WHtR values, 14.9% of male adolescents and 11% of female adolescents were classified as obese. The BF values indicate that 10.2% of male adolescents were overweight and 14.4% were obese, while 8.9% of female adolescents were overweight and 6.4% were obese. Regarding the differences in nutritional status, no significant differences were found among adolescents of both genders living in urban and rural areas in any of the examined variables.

**Table 3:** Differences in nutritional status among adolescents living in urban and rural areas

	Male							Female						
	**Urban**		**Rural**		**Overall**		**Chi**	**Urban**		**Rural**		**Overall**		**Chi**
	n	%	n	%	n	%	***p***	n	%	n	%	n	%	***p***
BMI														
Underweight	11	2.2	6	3	17	2.4	0.930	15	2.7	5	2.0	20	2.5	0.301
Normal	360	72.7	147	73.1	507	72.8		458	83.1	207	80.9	665	82.4	
Overweight	74	14.9	28	13.9	102	14.7		65	11.8	32	12.5	97	12.0	
Obese	50	10.1	20	10.0	70	10.1		13	2.4	12	4.7	25	3.1	
WHtR														
Normal	421	85.1	171	85.1	592	85.1	0.994	496	90.0	222	86.7	718	89.0	0.164
Obese	74	14.9	30	14.9	104	14.9		55	10.0	34	13.3	89	11.0	
Body Fat														
Underweight	79	16.0	42	20.9	121	17.4	0.442	11	2.0	13	5.1	24	3.0	0.053
Normal	292	59.0	112	55.7	404	58.0		461	83.7	198	77.3	659	81.7	
Overweight	50	10.1	21	10.4	71	10.2		44	8.0	28	10.9	72	8.9	
Obese	74	14.9	26	12.9	100	14.4		35	6.4	17	6.6	52	6.4	

Legend: BMI - Body Mass Index; WHtR - Waist to height ratio; Chi - Chi-squared test; *p* - significant value

Table 4 presents the results of physical activity levels among male adolescents and their differences by the place of residence. The mean physical activity score for males was 2.6 ± 0.7, while adolescents living in rural areas of Montenegro had a significantly higher level of physical activity compared to their counterparts in urban areas (2.7 ± 0.7; 2.6 ± 0.6; respectively, *p* = 0.032). Furthermore, adolescents living in rural areas exhibited significantly higher levels of physical activity during their spare time (1.6 ± 0.4; 1.5 ± 0.3; respectively, *p* = 0.002), physical education classes (3.6 ± 1.6; 3.2 ± 1.7; respectively, *p* = 0.002), little breaks (2.1 ± 1.0; 1.9 ± 0.8; respectively, *p* = 0.007), and lunchtime (2.1 ± 0.8; 2.0 ± 0.7; respectively, *p* = 0.034) compared to their peers in urban areas.

**Table 4:** Differences in level of physical activity among male adolescents living in urban and rural areas

PAQ-C Variables	Urban M ± SD	Rural M ± SD	Overall M ± SD	t-test	***p***
PA at spare time	1.5 ± 0.3	1.6 ± 0.4	1.5 ± 0.3	-3.14	0.002
PE class	3.2 ± 1.7	3.6 ± 1.6	3.3 ± 1.7	-3.16	0.002
Little break	1.9 ± 0.8	2.1 ± 1.0	2.0 ± 0.9	-2.70	0.007
Lunchtime	2.0 ± 0.7	2.1 ± 0.8	2.0 ± 0.8	-2.12	0.034
After school	3.1 ± 1.4	3.2 ± 1.3	3.1 ± 1.4	-1.11	0.268
Evening	2.9 ± 1.4	2.9 ± 1.4	2.9 ± 1.4	− 0.16	0.872
Weekend	3.0 ± 1.3	3.0 ± 1.3	3.0 ± 1.3	0.22	0.824
Statement that best describes PA	3.1 ± 1.2	3.1 ± 1.2	3.1 ± 1.2	0.61	0.544
FA by days	3.1 ± 1.1	3.1 ± 1.1	3.1 ± 1.1	0.19	0.850
Overall PAQ-C	2.6 ± 0.6	2.7 ± 0.7	2.6 ± 0.7	-2.15	0.032

Legend: PA -Physical activity; PE class - Physical education class; t-test - values for t-test; *p* - significant values

Table 5 presents the values of physical activity levels among female adolescents and their differences by the place of residence. The overall physical activity score was 2.2 ± 0.6, and no significant differences were found by the place of residence, except for spare time activity, where female adolescents living in rural areas were more physically active than their counterparts in urban areas (2.3 ± 0.6; 2.2 ± 0.6; respectively, *p* = 0.020).

**Table 5:** Differences in level of physical activity among female adolescents living in urban and rural areas

PAQ-C Variables	Urban M ± SD	Rural M ± SD	Overall M ± SD	t-test	***p***
PA at spare time	1.3 ± 0.3	1.4 ± 0.3	1.4 ± 0.3	-2.32	0.020
PE class	3.1 ± 1.6	3.2 ± 1.6	3.1 ± 1.6	-1.55	0.121
Little break	1.8 ± 0.7	1.9 ± 0.7	1.8 ± 0.7	− 0.97	0.333
Lunchtime	1.9 ± 0.6	1.9 ± 0.6	1.9 ± 0.6	− 0.02	0.982
After school	2.3 ± 1.3	2.4 ± 1.2	2.4 ± 1.3	− 0.64	0.519
Evening	2.3 ± 1.2	2.3 ± 1.2	2.3 ± 1.2	0.35	0.727
Weekend	2.3 ± 1.1	2.4 ± 1.0	2.3 ± 1.1	− 0.40	0.687
Statement that best describes PA	2.5 ± 1.0	2.6 ± 1.0	2.5 ± 1.0	− 0.86	0.389
FA by days	2.5 ± 0.9	2.6 ± 0.8	2.5 ± 0.9	-1.27	0.205
Overall PAQ-C	2.2 ± 0.6	2.3 ± 0.5	2.2 ± 0.6	-1.27	0.202

## Discussion

The aim of this study was to assess the status and differences in nutritional status and physical activity levels among adolescents living in urban and rural areas of Montenegro. Upon reviewing the results, it can be observed that differences in nutritional status did not exist for adolescents of both genders across any variable. However, when examining differences in the level of physical activity, distinctions were noted among male adolescents, with those residing in urban areas exhibiting statistically significantly lower levels of physical activity, while no differences were observed among their female counterparts.

Excluding differences related to place of residence and considering the overall nutritional status of adolescents in Montenegro, it can be observed that based on BMI values, 14.7% of male adolescents were classified as overweight and 10.1% as obese, while 12% of female adolescents were categorized as overweight and 3.1% as obese. According to WHtR values, 14.9% of male adolescents and 11% of female adolescents were classified as obese. Regarding BF values, 10.2% of male adolescents were identified as overweight and 14.4% as obese, while 8.9% of female adolescents were classified as overweight and 6.4% as obese. Comparing the percentage of overweight and obese adolescents in this study, which amounted to 24.8% for males and 15.1% for females based on BMI values, with the research conducted by Vasiljevic [7], where 15.2% of male adolescents and 9.7% of female adolescents in Montenegro were overweight or obese, it is evident that the results of Montenegrin adolescents have worsened compared to the year 2018. On the other hand, comparing these results with a study that included 15-year-old adolescents from 35 European countries [39], where is on average, 23% of male adolescents and 15% of female adolescents were classified as overweight or obese, it can be observed that adolescents from Montenegro have slightly higher values compared to their peers in Europe. According to the same study, if consider the surrounding countries where male (M) and female (F) adolescents in North Macedonia (M = 34%, F = 17%), Serbia (M = 28%, F = 15%), Albania (M = 28%, F = 10%), Slovenia (M = 26%, F = 16%), and Romania (M = 27%, F = 15%) were overweight or obese, it can be concluded that the prevalence of overweight and obesity among male adolescents in Montenegro is lower compared to neighboring countries, while it is approximately the same for female adolescents, excluding Albania where 10% of female adolescents were overweight or obese compared to 15% in Montenegro.

When examining the differences in nutritional status among adolescents living in urban and rural areas of both genders, it can be observed that there were no significant differences for male (*p* = 0.930) or female (*p* = 0.301) adolescents in terms of BMI, WHtR (*p* = 0.994; *p* = 0.164; respectively), or BF (*p* = 0.442; *p* = 0.053; respectively). Similar results have been found in studies involving adolescents from the United States [40], Kosovo [41], and Saudi Arabia [42]. In contrast to the results of this study, several other studies have reported that adolescents living in urban areas were statistically significantly more obese compared to their peers in rural areas, such as adolescents in China [27, 43], India [28], Malaysia [29], Austria [44], and others. Some possible reasons why adolescents in urban environments may be more obese include spending more time in front of TVs, computers, and phones [43], exhibiting higher levels of sedentary lifestyle, consuming higher fat intake through their diet [28], among other factors. On the other hand, some studies have shown that adolescents living in rural areas of Mexico [25], and Poland [26] were more obese compared to their counterparts in urban areas. Possible reasons for higher obesity rates among rural adolescents include consuming high-calorie food, similar access to phones, computers, and TV screens as their urban counterparts, lower levels of health education, and other factors [9].

The results of this study indicate that male adolescents had a moderate level of physical activity with a mean score of 2.6 ± 0.7, while female adolescents had a mean score of 2.2 ± 0.6. When comparing these results with adolescents from the United States [23], where male adolescents had a physical activity score of approximately 3.2 ± 0.7 and female adolescents had a score of 3.1 ± 0.7, it can be observed that adolescents from Montenegro had lower levels of physical activity compared to their peers from the US. On the other hand, male adolescents from Montenegro had similar levels of physical activity compared to their counterparts from China [37], with a score of 2.67 ± 0.7, while female adolescents had lower values compared to female peers whose physical activity score was 2.56 ± 0.7.

When considering the differences based on the place of residence, it can be seen that male adolescents living in rural areas of Montenegro (2.7 ± 0.7) had a statistically significant higher level of overall physical activity than their peers living in urban areas (2.6 ± 0.6) (*p* = 0.032). Furthermore, adolescents from rural areas had higher levels of physical activity than their urban peers during spare time (*p* = 0.002), physical education classes (*p* = 0.002), little breaks (*p* = 0.007), and lunchtime (*p* = 0.034). Similarly, female adolescents living in rural areas showed higher level of overall physical activity (2.3 ± 0.5) compared to their counterparts from urban areas (2.2 ± 0.6), although without statistical significance (*p* = 0.202). However, female adolescents from rural areas had a statistically significant higher level of physical activity only during their spare time (*p* = 0.020). Some reasons for lower levels of physical activity among adolescents in urban areas may include higher population density and limited space for engaging in informal sports activities, greater accessibility to transportation reducing their physical movement [26], more readily available and higher-quality internet access compared to peers in rural areas [45], among other factors. On the other hand, adolescents from rural areas may exhibit higher levels of physical activity due to engaging in daily physical tasks, which are particularly prevalent among the male population, as demonstrated in this study. Additionally, they spend more time outdoors [46]. Similar findings were observed in studies conducted in China [27], Malaysia [14, 29], and the United States [45], where individuals residing in rural areas demonstrated higher levels of physical activity or performed better in motor skill tests compared to their urban counterparts. However, some studies present different results. For instance, research conducted on Saudi Arabian [42], and Polish adolescents [26] revealed that urban adolescents exhibited higher levels of physical activity than their rural peers. Similarly, a study on Mexican adolescents [25] indicated that rural adolescents had a higher prevalence of sedentary lifestyle and performed worse on motor skill tests compared to their urban counterparts. The reason why adolescents from rural areas may exhibit lower levels of physical activity in some studies is that they often lack sufficient peers to engage in physical play, have fewer sports fields and centers to participate in physical activities, and have limited access to various sports clubs compared to adolescents in urban areas [42]. Additionally, the population in rural areas is increasingly reliant on motor vehicles, which is provided by better infrastructure in those areas, but can lead to decreased physical activity levels [24].

It is crucial to emphasize the significance of this study. This research represents the first national study in Montenegro that assesses differences in the nutritional status and level of physical activity among adolescents living in urban and rural areas. These innovative data can be highly valuable for formulating various strategies for the country of Montenegro. As there were no differences in nutritional status among adolescents living in urban and rural areas, a strategy addressing the issue of overweight and obesity is required equally in both. On the other hand, as male adolescents living in urban areas exhibited lower levels of physical activity compared to their peers in rural areas, special attention is needed to address this issue in urban areas. It could be recommended that future urban planning in Montenegro consider creating environments that encourage adolescents to engage in physical activity and adopt healthy lifestyles. It is significant to note that the results of this study revealed that male adolescents living in urban areas have significantly lower levels of physical activity during physical education classes, little breaks, and lunchtime. This underscores the necessity of creating environments that promote physical activity around schools, allowing urban adolescents to utilize their school breaks for play and other activities crucial for healthy growth and development. Such an environment would also enable physical education teachers to conduct outdoor classes, encouraging children’s activity during physical education classes [47]. In addition to creating environments that foster physical activity, there is a need for greater promotion of physical activity among adolescents in urban areas. Our study data show that both male and female adolescents are statistically less active during their spare time, suggesting that they may spend their free time watching TV, using computers, phones, and the like, which has long been a global trend.

However, this study has its limitations. This study did not collect data on how much time adolescents from rural areas spent in urban environments and vice versa, how much time adolescents living in urban environments spent in rural areas. This could also provide additional information on how different environments affect their lifestyle habits. Also, to assess the nutritional status, methods that might provide more accurate data, such as bioelectrical impedance, could have been used. Additionally, the study did not investigate the dietary habits of adolescents, which could provide valuable data regarding the issue of overweight and obesity. Also, it assesses the level of physical activity using a questionnaire, which, despite its proven validity and reliability when objectively completed, could still be subjectively filled and influenced by personal judgment, potentially reducing the validity of the obtained data. Another issue is that in the case of the questionnaire, adolescents living in rural areas who engage in daily physical tasks may not report that work as physical activity due to a misunderstanding of the concept, leading to the underreporting of their activity level [26]. Nevertheless, the mentioned limitations do not diminish the significance of this study, which has provided preliminary data on this topic in Montenegro on a sample of adolescents that can serve as an excellent starting point for future research. The limitations of this study may offer recommendations for future studies on this and similar subjects to obtain more precise data. Therefore, a recommendation for future research on this topic would be to use bioelectrical impedance for assessing nutritional status to obtain more precise data, and also to verify the dietary habits of the participants. The level of physical activity could be assessed using motor performance tests as employed in some studies [25, 27], or by using accelerometers (devices that work on a similar principle to GPS and estimate time spent in movement and sedentary behavior) [45], to obtain more valid data. Additionally, recommendations for future research could include measuring the same components in a sample of adults, as they significantly influence the lifestyles of their children, who often view them as their primary role models.

## Conclusion

The results of this study indicate that there were no differences in nutritional status among adolescents of both genders living in urban and rural areas of Montenegro. Therefore, the issue of overweight and obesity needs to be tackled progressively in both urban and rural areas. Furthermore, it is important to note that adolescents living in urban areas exhibited lower levels of physical activity (statistically significant for males, non-significant for females) compared to their counterparts in rural areas, highlighting the need for strategies to promote physical activity among adolescents in urban areas. It is necessary to pay attention to the mentioned aspects so that adolescents can have normal growth and development, and to reduce the issue of obesity and sedentary lifestyles.

## Data Availability

The datasets created and analyzed during the present study are available from the corresponding author upon reasonable request.
